# Generation of tunable Raman soliton and dispersive wave beyond 4 μm in centimeter-length fluorotellurite fibers

**DOI:** 10.1038/s41377-025-02045-z

**Published:** 2025-09-24

**Authors:** Juan Wang, Shunbin Wang, Xiabing Zhou, Mo Liu, Hao Wu, Yu Yin, Zhipeng Qin, Guoqiang Xie, Zhenrui Li, Pengfei Wang, Yichun Liu

**Affiliations:** 1 College of Physics and Optoelectronic Engineering, Harbin Engineering University, Harbin, 150001 China; 2 Qingdao Innovation and Development Center, Harbin Engineering University, Qingdao, 266400 China; 3 School of Physics and Astronomy, Key Laboratory for Laser Plasmas (Ministry of Education), Collaborative Innovation Center of IFSA (CICIFSA), Shanghai Jiao Tong University, Shanghai, 200240 China; 4 School of Physics, State Key Laboratory of Integrated Optoelectronics, Northeast Normal University, Changchun, 130022 China; 5 School of Physics, Key Laboratory of UV-Emitting Materials and Technology, Northeast Normal University, Changchun, 130022 China

**Keywords:** Fibre lasers, Solitons, Nonlinear optics

## Abstract

3–5-μm mid-infrared (MIR) ultrafast laser sources have garnered significant attention due to their critical applications in spectroscopy, environmental monitoring, and imaging. However, 4–5-μm compact fiber laser sources remain a significant technological challenge due to the lack of MIR fibers with good chemical stability, thermal stability, high nonlinearity, and low loss. Here, we develop fluorotellurite fibers based on 60TeO_2_-20BaF_2_-10AlF_3_-10Y_2_O_3_ (TBAY) glasses with a wide transmission window, demonstrating tunable Raman soliton and dispersive wave (DW) generation beyond 4 µm in centimeter-length fluorotellurite fibers pumped by a 3.54 μm femtosecond laser source. Fluorotellurite fibers with a loss of 0.39 dB/m were fabricated using a rod-in-tube method. The high numerical aperture (NA ~ 1.1@3.5 μm) of TBAY fibers allows the zero-dispersion wavelength (ZDW) to be tuned over a wide range by varying the core diameter of the fibers. The dispersion-engineered TBAY fibers with a core diameter of 6.5 μm enabled 4584 nm Raman soliton generation, while fibers with a core diameter of 3 μm enabled 4177 nm DW generation. We conducted detailed experiments to investigate the influence of pump power and fiber length on SSFS and dispersive wave dynamics. Theoretical analysis and numerical simulations based on the generalized nonlinear Schrödinger equation corroborate the experimental results. Our results show that TBAY fibers are promising nonlinear media for constructing compact ultrafast laser sources in the 4-5 μm wavelength range.

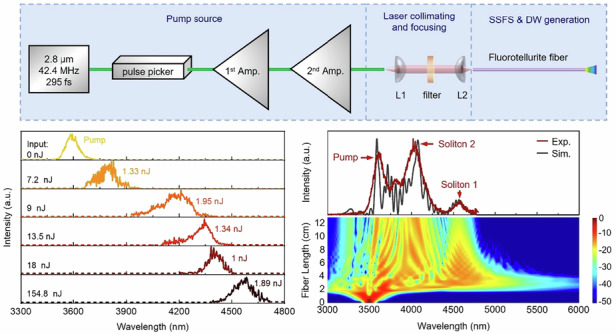

## Abstract (web summary)

Tunable 4584 nm Raman soliton and 4177 nm dispersive wave generation were demonstrated in centimeter-lengths, dispersion-engineered fluorotellurite fibers pumped by a 3.54 µm laser.

## Introduction

Mid-infrared (MIR) ultrafast laser sources have garnered significant attention due to their critical applications in spectroscopy^1,2^, environmental monitoring^3,4^, and two-photon fluorescence imaging^5,6^. In particular, coherent light sources beyond 4 μm open new avenues for exploring molecular vibrations and other phenomena unique to this wavelength range^7,8^. MIR fiber lasers are primarily based on two mechanisms: rare earth ion-doped fiber amplification and fiber nonlinear effects^9,10^. Laser emission around 3–4 µm can be achieved using Dy^3+^, Ho^3+^, and Er^3+^-doped fluoride fibers^11–14^. However, achieving lasing at wavelengths longer than 4 µm with rare earth ion-doped fibers is challenging due to the dominance of nonradiative transitions in the process^15^. Therefore, mechanisms based on fiber nonlinear effects are more effective for generating lasing at wavelengths beyond 4 µm.

Raman soliton and dispersive wave (DW) generation in optical fibers, as promising methods for obtaining fiber-based mid-infrared light sources, have been widely investigated. Raman soliton generation is induced by Raman gain, where the blue portion of the soliton spectrum pumps the red portion, causing a continuous red shift in the spectrum, known as soliton self-frequency shift (SSFS)^16–18^. Interestingly, SSFS can be canceled by a negative dispersion slope, allowing for the generation of tunable red-shifted dispersive waves in fiber^19^. To generate MIR Raman soliton or DW laser sources, significant efforts have been made to develop non-silica fibers, such as tellurite, fluoride, and chalcogenide fibers, with low transmission loss in the MIR region^20–23^. So far, 4–5 μm Raman soliton and DW laser sources have only been achieved using fluoroindate (InF_3_) fibers as the nonlinear medium. Tang et al. reported a fiber-based system that generates 100 fs pulses with 5 nJ energy, continuously wavelength-tunable over 2–4.3 µm through the SSFS in a 2-m long InF_3_ fiber^24^. Recently, Gauthier et al. experimentally demonstrate SSFS tuning of femtosecond pulses up to 4.8 µm in a 20 m long InF_3_ fiber^25^. In the InF_3_ fiber-based laser system, long fiber (several meters or tens of meters) is required due to the weak nonlinear refractive index of InF_3_ glass, which limits the compactness of laser systems. On the other hand, Bei et al. investigated the chemical durability of InF_3_ glass by leaching glass samples in deionized water^26^. The absorption due to OH- stretching and HOH bending vibrations in InF_3_ glass increased with the amount of hydrated layers formed during leaching. An endcap should be used to protect the InF_3_ fiber tip from potential optical or thermal damage when developing high-power MIR laser sources^27^. These results show that the poor chemical and thermal stability of InF_3_ glasses limits their practical application. Despite recent progress in this field, exploring novel fiber materials with good chemical and thermal stability is essential for realizing 4–5 μm Raman soliton and DW laser sources.

In this Letter, we report the development of fluorotellurite fibers based on TBAY glasses with a wide transmission window and good chemical and thermal stability, demonstrating tunable Raman soliton and DW generation beyond 4 µm in TBAY fibers pumped by a 3.54 μm femtosecond laser source. The dispersion-engineered TBAY fibers with a core diameter of 6.5 μm enabled Raman soliton generation at 4584 nm, while those with a core diameter of 3 μm enabled DW generation at 4177 nm. Our results demonstrate that TBAY fibers are promising nonlinear media for the development of ultrafast laser sources beyond 4 μm.

## Results

### Characteristics of glass

**Fig. 1:**
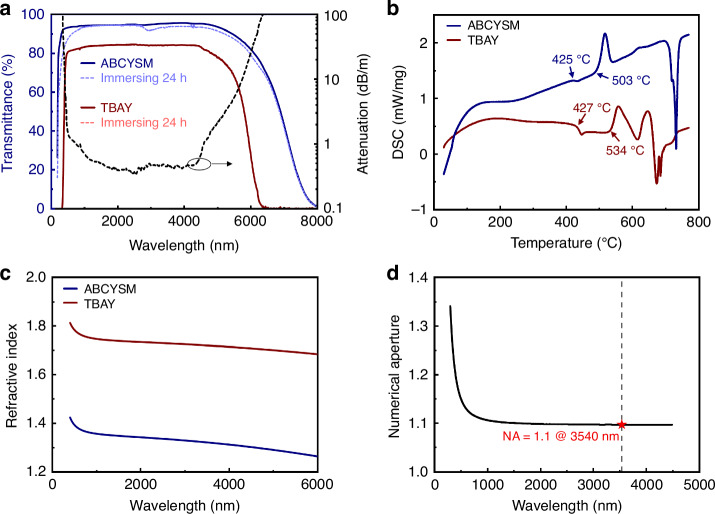
Characteristics of TBAY and ABCYSM glass. **a** Transmittance spectrum before and after immersing ultrapure water experiment and calculated core attenuation curve. **b** DTA curves. **c** Refractive index. **d** Numerical aperture

The transmission spectra of the TBAY (60TeO_2_-20BaF_2_-10AlF_3_-10Y_2_O_3_) and ABCYSM (35AlF_3_-10BaF_2_-20CaF_2_-15YF_3_-10SrF_2_-10MgF_2_) glasses are depicted in Fig. 1a, represented by the blue and red solid curves, respectively. These two types of glass exhibit high transmittance within the wavelength range of 0.5–6 μm. The aforementioned results demonstrate that the incorporation of BaF_2_ and AlF_3_ significantly reduced the hydroxyl group content within the glass matrix. To evaluate the water resistance of TBAY and ABCYSM glasses, the transmission spectra of the cladding and core glasses were measured after immersion in ultrapure water for 24 h. The results are illustrated in Fig. 1a, with the red and blue dashed curves representing the TBAY and ABCYSM glasses, respectively. No significant changes were observed in the transmission spectra, indicating that both TBAY and ABCYSM glasses exhibit excellent water resistance. The core attenuation curve of the TBAY fiber from 0.5 to 6 μm was also calculated from glass transmittance, the thickness of TBAY glass sample is 0.2 mm, as shown in Fig. 1a. It is noticeable that beyond 4.3 μm, the core attenuation exhibits a significant increase resulting from the multiphonon absorption edge effect, which is an intrinsic material constraint^28^. Multiple phonons are created in conjunction with the absorption of a single photon during absorption processes. They can lead to substantial absorption tails in spectral regions where one would otherwise have low absorption. The contributed absorption decays exponentially towards shorter optical wavelengths, as increasingly higher-order processes are required in that spectral region. The temperature dependence of elongation (ΔL) for TBAY and ABCYSM glass rods of identical length (~0.2 cm) is presented in Fig. 1b. Both glasses exhibited similar glass transition temperatures (T_g_) and onset crystallization temperatures (T_x_). Specifically, the T_g_ and T_x_ values of TBAY glass were 427 °C and 534 °C, respectively, while those of ABCYSM glass were 425 °C and 503 °C, respectively. These results demonstrate that step-index fluorotellurite fibers can be successfully fabricated utilizing TBAY glass as the core material and ABCYSM glass as the cladding material. The measured refractive indices of TBAY and ABCYSM glasses are presented in Fig. 1c. The refractive index difference between ABCYSM and TBAY glasses is significantly large, enabling the fabrication of high numerical aperture (NA) fluorotellurite fibers. As illustrated in Fig. 1d, the calculated NA of fluorotellurite fibers based on these two glasses reaches ~1.1 at 3.54 μm. This high NA value provides exceptional dispersion design flexibility for the fibers.

### Design of fiber

**Fig. 2:**
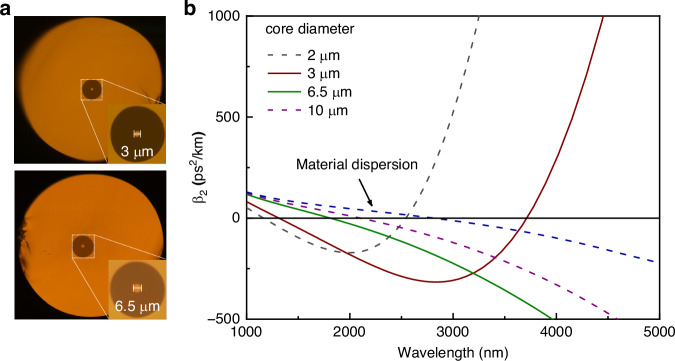
Microstructure and dispersion characteristics of the TBAY fiber. **a** The microscope image of the TBAY fiber sample. **b** Calculated dispersion curves for different core diameters

The cross section of the fabricated high numerical aperture (NA) fluorotellurite fiber is presented in Fig. 2a, revealing a step-index structure. The background loss of the fibers was ~0.39 dB/m at 3.54 µm, as determined by the cut-back method. The calculated group velocity dispersion (GVD) curves of the fundamental propagation mode for the TBAY fiber with core diameters of 10, 6.5, 3.0, and 2.0 µm are presented in Fig. 2b. The first zero-dispersion wavelengths (ZDWs) for these core diameters were 2102 nm, 1805 nm, 1315 nm, and 1162 nm, respectively. Additionally, the second ZDWs for fibers with core diameters of 3.0, and 2.0 µm were 3723 nm, and 2540 nm, respectively. The nonlinear coefficients at 3.54 μm for the corresponding core diameters were calculated to be 15.47, 22.91, 86.15, and 98.67 km⁻¹ W⁻¹, respectively, using a nonlinear refractive index of 3.5 × 10⁻^19^ m² W⁻¹ for fluorotellurite glasses^21,22^. Generally, the generation of MIR Raman solitons requires fluorotellurite fibers with a high nonlinear coefficient and anomalous dispersion at the pumping wavelength (∼3.54 μm). For the generation of MIR dispersive waves (DWs), fibers with a negative dispersion slope at MIR wavelengths (>3.5 μm) are essential. Based on these requirements, a fluorotellurite fiber with a core diameter of 6.5 µm was selected as the nonlinear medium for generating MIR Raman solitons, while a fiber with a core diameter of 3.0 µm was used for generating MIR DWs in our experiments.

### Experimental setup

**Fig. 3:**
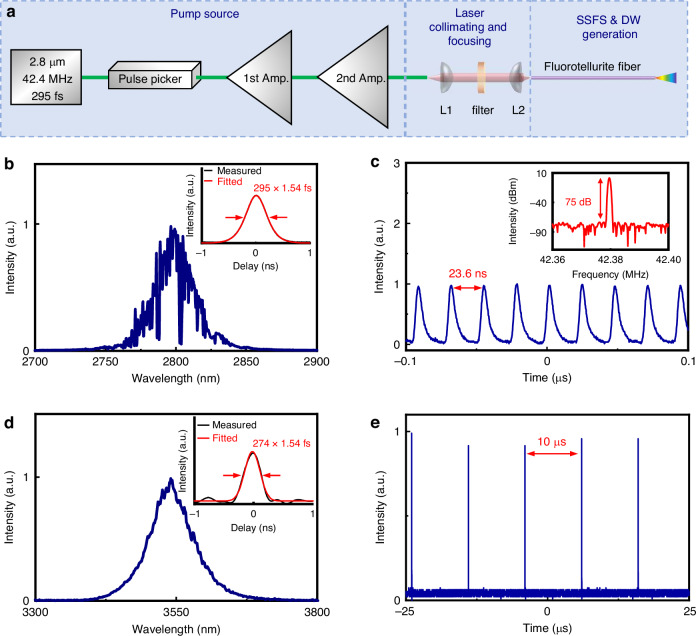
Experimental setup and output characteristics of the oscillator and 2^nd^ amplifier. **a** Experimental setup. **b** Optical spectrum (inset: pulse duration) and **c** Oscilloscope traces (inset: RF signal to noise ratio) of oscillator. **d** Optical spectrum (inset: pulse duration) and **e** Oscilloscope traces of 2^nd^ amplifier

To demonstrate the potential of the fluorotellurite fiber for generating tunable MIR Raman solitons and DWs, we conducted the following experiments. The experimental setup is illustrated in Fig. 3a. The pump source comprises a 2.8 μm seed laser and two-stage amplifier. The 2.8 μm seed laser is a passively mode-locked ring laser incorporating a ~3 m segment of Er^3+^-doped fluoride fiber. The mode-locking system, based on nonlinear polarization evolution (NPE) technology, generates 295 fs pulses with a pulse energy of 2.4 nJ, corresponding to a peak power of ∼8.14 kW. The emission spectrum of the seed laser, measured using a FTIR spectrum analyzer (Thermo, Nicolet iS50), is presented in Fig. 3b. The inset of Fig. 3b displays the autocorrelation trace of the seed laser, obtained using an autocorrelator. The pulse train of the seed laser is depicted in Fig. 3c, with a pulse interval of 23.6 ns, corresponding to a repetition rate of 42.4 MHz. The inset of Fig. 3c displays the radio frequency (RF) spectrum measured by an oscilloscope (Tektronix, MDO3102) with a photodetector (VIGO System, MIP-10k-100M-F-M4). The fundamental frequency of the mode-locked pulse is 42.4 MHz with a signal-to-noise (SNR) of 75 dB, indicating excellent stability. Subsequently, a pulse picker reduces the repetition rate from 42.4 MHz to 100 kHz to optimize pulse energy management. The pulses, after repetition rate adjustment and first amplification, undergo a second amplification stage. During this stage, the central wavelength of the pulses shifts from ~2.8 μm to ~3.54 μm due to the Raman soliton self-frequency shift effect in the fluoride fiber amplifier. The laser beam is then collimated by an aspheric ZnSe lens L1 (f = 20 mm) and passes through a filter to refine the spectral profile. In the second stage, the filtered ultrashort pulse centered at 3540 nm is focused into the TBAY fiber using an aspheric Black Diamond-2 lens L2 (f = 4 mm). After the second-stage amplifier, the maximum pulse energy of the filtered 3.54 μm pulse reaches 260 nJ. The measured spectrum of the 3540 nm laser is shown in Fig. 3d, with the inset displaying the autocorrelation trace, revealing a pulse width of 274 fs. Figure 3e illustrates the pulse train of the 3540 nm femtosecond laser, with a pulse interval of 10 μs, corresponding to a repetition rate of 100 kHz.

### SSFS in TBAY fiber

**Fig. 4:**
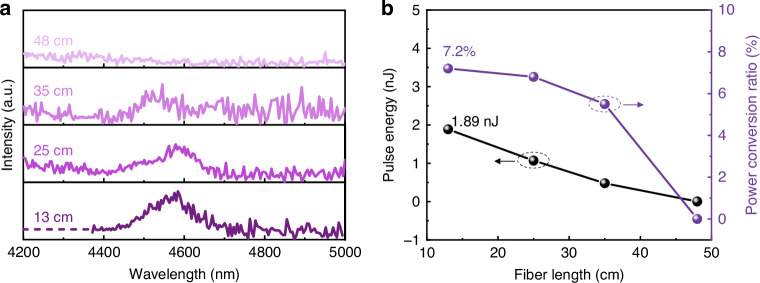
Output characteristics of fibers with different lengths at an incident pulse energy of 154.8 nJ. **a** Output spectrums. **b** Output pulse energy and conversion efficiency for the first Raman soliton

In the experiments, a 48-cm-long TBAY fiber was initially utilized, the spectrum performance of the TBAY fiber is measured using a spectrum analyzer (Fastlite, Mozza). However, the coupling efficiency of the pump pulse into the TBAY fiber was relatively low, estimated at ~25.8%, due to the small core size and high Fresnel reflection (~8%) at the fiber ends. To investigate the influence of fiber length on the soliton self-frequency shift, the cut-back method was employed. This involved gradually reducing the length of the TBAY fiber while monitoring the evolution of the Raman soliton. Figure 4a presents the measured output spectra for different fiber lengths under an incident pulse energy of 154.8 nJ. The spectral characteristics and power of the generated soliton were meticulously monitored as the fiber length was progressively reduced. When the fiber length was 48 cm, the SSFS phenomenon was clearly observed. As the fiber length was decreased from 48 cm to 13 cm, the intensity of the first Raman soliton centered at 4584 nm gradually increases due to the reduction of wavelength-dependent fiber-loss. For a fiber length of 13 cm, the maximum output pulse energy of the first Raman soliton was estimated to be 1.89 nJ, with a conversion efficiency of 7.2%, as illustrated in Fig. 4b. However, it is noteworthy that the 4.6 μm soliton was not observed in the 48-cm-long fiber, while it became detectable in the shorter fiber setup. This can be attributed to two primary mechanisms. First, the wavelength-dependent loss in the TBAY fiber becomes increasingly significant at longer wavelengths, particularly around 4.6 μm, where the material absorption is substantially higher, as shown in Fig. 1a. In the 48 cm fiber, this enhanced attenuation suppresses the 4.6 μm soliton component, making it below the detection threshold. Second, the high-order soliton undergoes temporal splitting and energy redistribution among multiple spectral components. This process disperses the available energy across a broader wavelength range, consequently reducing the power fraction allocated to the specific 4.6 μm soliton. The combination of these two effects results in the 4.6 μm soliton having an extremely low power ratio in longer fibers, making it practically undetectable. Conversely, the reduced fiber length of 13 cm reduces both the loss effects and the extent of soliton fission, thereby preserving sufficient power in the 4.6 μm spectral region for experimental observation.

**Fig. 5:**
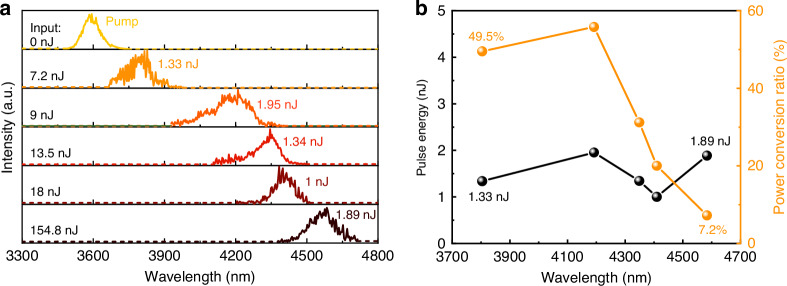
Output characteristics of 6.5 μm core diameter TBAY fibers. **a** Measured output spectrum for 13-cm-long fiber at different incident pulse energy. **b** Measured soliton conversion efficiency and pulse energy for the first Raman soliton

Figure 5a shows the dependence of the measured output spectra from the 13 cm long fluorotellurite fiber on the incident pulse energy of the 3540 nm femtosecond fiber laser. The GVD value at 3540 nm of the fiber was about −73.61 ps^2^/km and the calculated dispersion length L_D_ was ~25 cm. For the soliton A(0, T) = N sech (T/T_0_), the soliton order N of the input pulse is determined by both the pulse and fiber parameters through N^2^ = L_D_/L_NL_, where L_NL_ is nonlinear length^10^. The soliton fission length is defined as L_fiss_ = L_D_/N. For the 13 cm long fluorotellurite fiber, the first Raman soliton with a central wavelength of ~3804 nm was generated by the soliton fission as the incident pulse energy reached 7.2 nJ (In this case, L_fiss_ = 1.38 cm). As the incident pulse energy was increased to ~9 nJ, the SSFS phenomenon became clearly observable, with the first Raman soliton redshifted to ~4192 nm due to intra-pulse Raman scattering. When the incident pulse energy was further increased to 13.5 nJ, the Raman soliton redshifted to ~4349 nm. The first soliton can be frequency-shifted to 4584 nm at an incident pulse energy of 106.6 nJ and maintains its central wavelength until the pulse energy reaches 154.8 nJ. Figure 5b illustrated the output pulse energy and conversion efficiency for first Raman solitons at different wavelengths in the 13-cm-long fluorotellurite fiber. The conversion efficiency for the generated 4584 nm Raman soliton was ~7.2%. The increased material loss and soliton fission-induced energy transfer leads to a significantly decreased conversion efficiency of first Raman soliton. These results demonstrate that tunable MIR Raman solitons, spanning from 3540 nm to 4584 nm, can be achieved in the fluorotellurite fiber through the SSFS mechanism. To the best of our knowledge, this study represents the first demonstration of tunable Raman soliton generation beyond 4.5 μm in fluorotellurite fibers.

**Fig. 6:**
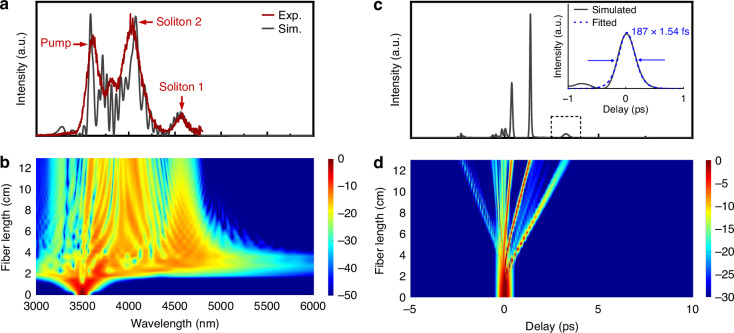
Simulated output characteristics of 6.5 μm core diameter TBAY fibers at incident pulse energy of 154.8 nJ. **a** Measured (red) and simulated (gray) output spectrum. **b** Spectrum evolution. **c** Simulated pulse propagation, inset: simulated pulse duration of 4.6 μm Raman soliton. **d** Temporal evolution

Additionally, we conducted numerical simulations of MIR Raman soliton generation in fluorotellurite fibers by solving the generalized nonlinear Schrödinger equation (GNLSE). In the simulations, the following parameters were used: a calculated nonlinear coefficient of 22.91 km⁻¹ W⁻¹; the chromatic dispersion data shown in Fig. 2b; a pump laser with an operating wavelength of ~3540 nm, a pulse width of ~274 fs, and a repetition rate of 100 kHz; and the Raman response function derived from the Raman gain spectrum of the fluorotellurite glass. The Raman response function was characterized by dual time constants of *τ*_*1*_ = 7.03 fs and *τ*_*2*_ = 65.18 fs, with a fractional Raman contribution *f*_*R*_ = 0.32 for the TBAY glass composition. These parameters were determined from the intermediate-broadening model to accurately represent the vibrational dynamics of the TBAY glass^29^ (See Supplementary Information). To account for wavelength-dependent transmission characteristics, the simulations incorporated the measured attenuation profile illustrated in Fig. 1a. Figure 6a presents a comparison between the simulated (gray curve) and measured (red curve) Raman soliton spectra output from the fluorotellurite fiber under the same incident pulse energy of 154.8 nJ. The close agreement between the simulated and experimental results confirms the appropriateness of the parameters used in the numerical simulations. The experimentally observed three spectral peaks at 3.6 μm, 4.0 μm, and 4.6 μm exhibit characteristic hyperbolic secant function-based distribution profiles (See Supplementary Information), confirming their origin as fundamental Raman solitons generated through higher-order soliton fission and subsequent self-frequency shift. The spectral and temporal evolution of Raman soliton generation in the fluorotellurite fiber for an incident pulse energy of 154.8 nJ is shown in Fig. 6b, d. As shown in Fig. 6b, the initial spectral broadening in the Raman soliton evolution was caused by higher-order soliton compression as the pump pulse propagated through the fiber segment from 0 to 2 cm. For the pump pulse propagating through the fiber segment from 2 to 5 cm, further spectral broadening was observed. Since the operating wavelength of the pumping laser was located in the anomalous dispersion region of the fluorotellurite fiber, we considered that the mechanisms of large spectral broadening were a combination of higher order soliton compression, soliton fission, and the soliton self-frequency shift. As the pump pulse propagated through the fiber segment from 5 to 13 cm, the Raman soliton further redshifted to 4584 nm due to intra-pulse Raman scattering. Figure 6c illustrated the simulated pulse propagation in 13-cm-long TBAY fiber, and the simulated pulse duration of the obtained 4.6 μm Raman soliton is shown in the inset, with a pulse duration of 187 fs and an estimated peak power of 10.1 kW, loss-corrected theoretical net output pulse energy and peak power are 2.12 nJ and 11.34 kW, respectively. The temporal evolution shows in Fig. 6d corresponds well to the spectrum evolution, indicating the reliability of our simulation. Although direct measurement of the Raman soliton pulse duration was difficult due to limited output power, simulations indicate sub-picosecond durations consistent with the measured spectra. To enable full temporal characterization in future work, strategies such as pump pre-amplification using mid-IR fiber amplifiers^30^, and integration of Pr^3+^ or Dy^3+^-doped fiber amplifiers are promising^31,32^. Additionally, coupling efficiency can be improved via optimized alignment, mode-matching optics, and anti-reflection coatings. These enhancements could increase soliton output power sufficiently for autocorrelation or FROG measurements, advancing the practical development of compact ultrafast sources beyond 4 μm.

### DW generation

**Fig. 7:**
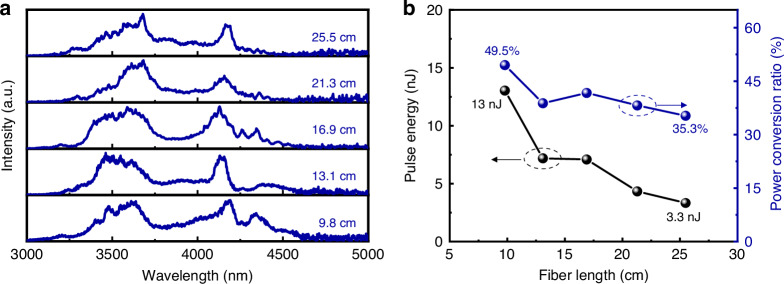
Output characteristics of 3 μm core diameter TBAY fibers. **a** Measured optical spectrum at different fiber length with the incident pulse energy of 260 nJ. **b** Output power and conversion efficiency

In our experiments, a fluorotellurite fiber with a core diameter of 3 μm was employed for generating MIR DWs. The coupling efficiency of the pump pulse into the 3-μm core TBAY fiber was estimated to be ~10.8%. Figure 7a presents the measured optical spectra at different fiber lengths. The initial experiment utilized a 25.5-cm-long TBAY fiber. When the incident pulse energy was increased to 220 nJ a wavelength component near 4177 nm was observed. To investigate the influence of fiber length, the fiber was progressively cut to shorter lengths of 21.3 cm, 16.9 cm, 13.1 cm, and 9.8 cm. For each length, the experimental observations were consistent with those from the 25.5-cm-long fiber: a spectral component near 4177 nm emerged at low pump powers, and no significant wavelength shifts were observed as the pump power increased. As the fiber length was reduced from 13.1 cm to 9.8 cm, the Raman soliton further transferred energy to a redshifted dispersive wave DW. The dip observed at 4250 nm is attributed to CO_2_ absorption. Figure 7b illustrates the output pulse energy and power ratio of DWs for different fiber lengths. For the 9.8-cm-long fiber, the maximum output pulse energy and power ratio of the generated DW were ~13 nJ and 49.5%, respectively. We attribute the enhanced DW generation in shorter fibers to the reduced infrared absorption loss at wavelengths beyond 4 μm.

**Fig. 8:**
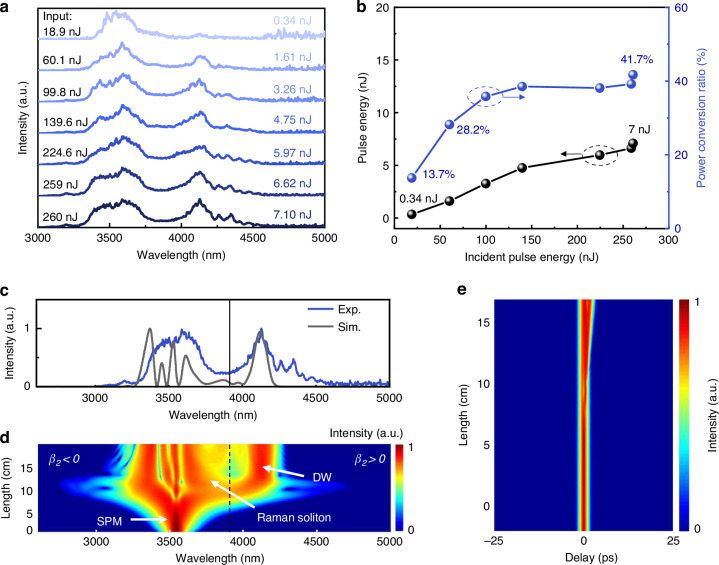
Output characteristics of 16.9-cm-long TBAY fiber. **a** Measured optical spectrum at different incident pulse energy. **b** Output pulse energy and conversion efficiency as a function of incident pulse energy. **c** Simulated (gray) and measured (blue) output spectrum at the incident pulse energy of 260 nJ. **d** Simulated spectral evolution of output signals at the incident pulse energy of 260 nJ. **e** Simulated temporal evolution of output signals at the incident pulse energy of 260 nJ

Figure 8a illustrates the dependence of the output spectra from the 16.9-cm-long TBAY fiber on the incident pulse energy. As the incident pulse energy was increased from 18.9 nJ to 60.1 nJ, the DW was observed at 4177 nm. Since the pump laser wavelength was located in the anomalous dispersion region of the fluorotellurite fiber, the generation of DWs can be attributed to a combination of higher-order soliton compression, soliton fission, SSFS and the SSFS cancellation effect. When the incident pulse energy was further increased from 60.1 nJ to 260 nJ, the conversion efficiency and output pulse energy of the DWs increased to 41.7% and 7 nJ, respectively, as shown in Fig. 8b. The loss-corrected theoretical net output pulse energy and peak power is 7.9 nJ and 68.7 kW, respectively. The dual-peak spectral structure observed in Fig. 8a arises from the dynamic balance between soliton self-frequency shift toward longer wavelengths and dispersive wave generation that creates complementary blue-shifted components through energy-conserving soliton recoil. The broadband supercontinuum formed by pump and soliton overlap contributing 58.3% power.

To verify the mechanism for the generation of the 4177 nm dispersive wave, we performed numerical simulations by solving the generalized nonlinear Schrödinger equations, the spectral and temporal evolution of MIR dispersive waves generation in the dispersion-engineered fluorotellurite fibers was simulated. The dispersion data of TBAY fiber with 3-μm core diameter is from Fig. 2b, the pump conditions are the same as to those in SSFS, the incident pulse energy is set to 260 nJ. Figure 8c presents a comparison between the theoretically simulated and experimentally measured spectra, demonstrating excellent agreement. The spectral evolution shows in Fig. 8d indicate that, since the wavelength of the pump laser was located at the anomalous dispersion region of the fluorotellurite fiber, the mechanisms of DW generation is combination of higher-order soliton compression, soliton fission, Raman soliton self-frequency shift. Interestingly, as the red-shifted soliton moves into the spectral region in which the dispersion slope of the dispersion engineered fiber with two ZDWs is negative, the soliton would emit a radiation band with a wavelength of longer than the second ZDW through the Cherenkov mechanism, the Cherenkov radiation is also called red-shifted dispersive wave. This triggers energy transfer from the soliton to the DW, visible as a sharp spectral peak at 4177 nm. The corresponding temporal evolution of DW generation was shown in Fig. 8e, which confirmed the above interpretation.

## Discussion

**Fig. 9:**
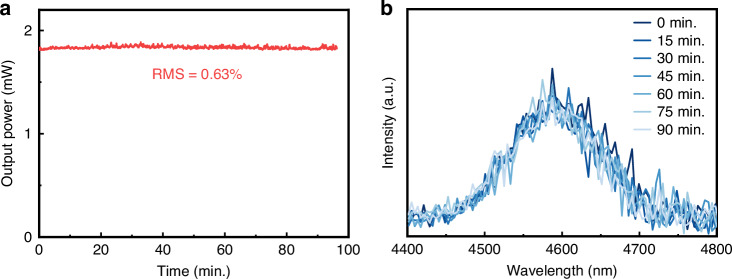
Long-term output power and spectral stability. **a** Output power stability over 90 minutes. **b** Spectral stability and 90 minutes

Finally, we compared the performance of this work with other ultrashort pulse fiber lasers operating in the 4–5-μm wavelength range. Table 1 summarizes recent advances in gain medium characteristics and key performance metrics of 4–5 μm ultrashort pulse fiber lasers. Compared with InF_3_-based systems, TBAY fiber enables significantly shorter device length of 13 cm due to its higher nonlinearity, while maintaining stable 4.6 μm output (1.82 mW, 0.63% RMS) over 90 min, demonstrating superior output stability, as illustrated in Fig. 9a. Information on output repeatability can be found in the Supplementary Information. Within the available pump power range, the fiber end face was not damaged (See Supplementary Information). Our developed TBAY fiber holds significant potential for generating compact, high-energy 4–5 μm Raman solitons and dispersive waves. The MIR laser source demonstrated in this study exhibits great promise for cross-disciplinary applications, including trace gas detection, organic material processing, biomedical research, and other fields.

**Table 1:** Recent advances in gain medium characteristics and key performance metrics of 4–5-μm ultrashort pulse fiber lasers

References	Fiber material	Mechanism	Working wavelength	Output RMS	Fiber length	n_2_ (m^2^ W^−1^)	T_g_ (°C)	Water resistance
24	InF_3_	SSFS	4.3 μm	/	2 m	4.0 × 10^−20^	~309^33^	Poor^26^
25	InF_3_	SSFS	4.8 μm	/	20 m	2.1 × 10^−20^	~309^33^	Poor^26^
This work	TBAY	SSFS & DW	4.6 μm & 4.1 μm	0.63%	0.13 m	3.5 × 10^−19^	427	Good

In summary, we have developed a novel TBAY glass with a broadband infrared transmission window, excellent thermal stability, chemical stability, and high nonlinearity. By carefully controlling dispersion and optimizing nonlinear processes, we successfully generated Raman solitons and dispersive waves with wavelengths exceeding 4 μm in centimeter-length TBAY fibers. The dispersion-engineered TBAY fibers with a core diameter of 6.5 μm enabled the generation of 4584 nm Raman solitons, while a core diameter of 3 μm facilitated the generation of 4177 nm DW. Detailed experiments were conducted to investigate the influence of pump power and fiber length on SSFS and DW dynamics. However, further reductions in fiber loss and improvements in power handling are necessary to extend the tuning range and increase output power. Future work should focus on optimizing fiber composition to improve nonlinearity and reduce losses, as well as exploring polarization-maintaining designs and advanced pumping schemes. Our results demonstrate that TBAY fibers are highly promising nonlinear media for generating MIR Raman solitons and DWs beyond 4 μm. This study provides a solid foundation for the development of integrated MIR fiber sources and enhances our understanding of nonlinear optics in this critical spectral region.

## Materials and methods

### Preparation of glass materials

The cladding and core materials of the fluorotellurite fibers were 35AlF_3_-10BaF_2_-20CaF_2_-15YF_3_-10SrF_2_-10MgF_2_ (ABCYSM) and 60TeO_2_-20BaF_2_-10AlF_3_-10Y_2_O_3_ (TBAY) glasses, respectively. In the core material, BaF_2_ was added to reduce the hydroxyl group content, as F⁻ ions can break the O–H bonds in the glass. AlF_3_ was incorporated to further enhance the removal of hydroxyl groups and to adjust the refractive index of the glass. Additionally, Y_2_O_3_ was included to improve the thermal stability of the glass, preventing crystallization during fiber drawing. In our experiments, starting chemical materials with a purity of 99.99% were used. First, bulk glass samples of TBAY and ABCYSM were prepared using a melting method. The glass samples were polished to a thickness of 2 mm for optical measurements. The transmission spectra from ultraviolet to mid-infrared were recorded using a Perkin-Elmer Lambda 750 UV–VIS–NIR spectrophotometer (190–3300 nm) and a Perkin-Elmer FTIR spectrometer (2500–25000 nm), respectively. The refractive indices of the glass samples were characterized using a spectroscopic ellipsometer (SE850, Sentech). Thermal properties were determined by differential thermal analysis (DTA) with a PerkinElmer TG-DTA6200 analyzer at a heating rate of 10 °C/min in the temperature range of 25–770 °C.

### Fabrication of fiber

The fibers were fabricated using the rod-in-tube method and feature a single-cladding structure. First, the core and cladding materials were melted at 950 °C under a dry atmosphere composed of 90% nitrogen and 10% oxygen. The molten materials were then poured into a preheated brass mold to form the preform. Next, the preform was heated and drawn into a ‘rod’ structure, consisting of a TBAY core glass surrounded by ABCYSM cladding glass. Simultaneously, the ‘tube’ (ABCYSM glass) was produced using the rotational casting method. Finally, the thinner rod was inserted into an ABCYSM glass tube and drawn into fibers.

To enhance optical quality and system performance, several fabrication optimizations were implemented, including upgrading from 4N to 5N purity raw materials for both TBAY core (60TeO_2_-20BaF_2_-10AlF_3_-10Y_2_O_3_) and ABCYSM cladding (35AlF_3_-10BaF_2_-20CaF_2_-15YF_3_-10SrF_2_-10MgF_2_) glasses with sealed crucible melting and enhanced stirring to reduce transition metal and hydroxyl contamination. During preform processing, we replaced deionized water with anhydrous ethanol for moisture-sensitive ABCYSM polishing to ensure precise geometric control, while fiber drawing employed 99.999% high-purity Ar atmosphere instead of nitrogen to achieve lower dew points and minimize hydroxyl absorption during controlled temperature and tension processes.

### Supplementary information

Supplementary information for the calculation of Raman response function, output repeatability of 4.6 μm Raman soliton, damage threshold of TBAY fiber and hyperbolic secant function-based optical soliton spectrum fitting accompanies the manuscript on the Light: Science & Applications website (http://www.nature.com/lsa).

## Supplementary information

Supplementary Information for Generation of Tunable Raman Soliton and Dispersive Wave Beyond 4 μm in Centimeter-Length Fluorotellurite Fibers

## Data Availability

Data underlying the results presented in this paper are not publicly available at this time but may be obtained from the authors upon reasonable request.

## Code availability

All custom codes used in this study are available from the corresponding authors upon reasonable request.

## References

[1] Hall, J. L. et al. Ultrasensitive spectroscopy, the ultrastable lasers, the ultrafast lasers, and the seriously nonlinear fiber: a new alliance for physics and metrology. *IEEE J. Quantum Electron.* **37**, 1482–1492 (2001).

[2] Chang, G. Q. & Wei, Z. Y. Ultrafast fiber lasers: an expanding versatile toolbox. *iScience* **23**, 101101 (2020). 32408170 10.1016/j.isci.2020.101101 PMC7225726

[3] Lu, M. J. et al. Interpulse stimulation Fourier-transform coherent anti-Stokes Raman spectroscopy. *Photonics Res.* **11**, 357–363 (2023).

[4] Webber, M. E., Pushkarsky, M. & Patel, C. K. N. Optical detection of chemical warfare agents and toxic industrial chemicals: Simulation. *J. Appl. Phys.* **97**, 113101 (2005).

[5] Srinivasan, T. & Yildirim, M. Advances in ultrafast fiber lasers for multiphoton microscopy in neuroscience. *Photonics* **10**, 1307 (2023).

[6] Chou, L. T. et al. Compact multicolor two-photon fluorescence microscopy enabled by tailorable continuum generation from self-phase modulation and dispersive wave generation. *Opt. Express* **30**, 40315–40327 (2022). 36298966 10.1364/OE.470602

[7] Shamim, M. H. M. et al. All-fiber coherent supercontinuum generation in a cascade of silica, fluoride, and chalcogenide fibers. *J. Phys. Photonics* **6**, 045018 (2024).

[8] Lesko, D. M. B. et al. A six-octave optical frequency comb from a scalable few-cycle erbium fibre laser. *Nat. Photonics* **15**, 281–286 (2021).

[9] Jackson, S. D. Mid-infrared fiber laser research: tasks completed and the tasks ahead. *APL Photonics* **9**, 070904 (2024).

[10] Agrawal, G. *Nonlinear Fiber Optics*, 5th edn. (Academic Press, 2013).

[11] Jackson, S. D. Continuous wave 2.9 μm dysprosium-doped fluoride fiber laser. *Appl. Phys. Lett.* **83**, 1316–1318 (2003).

[12] Zhang, J. et al. ZnF_2_-modified AlF_3_-based fluoride glasses with enhanced mid-infrared 3.5 μm emission. *J. Am. Ceram. Soc.* **105**, 4691–4698 (2022).

[13] Schneide, J., Carbonnier, C. & Unrau, U. B. Characterization of a Ho^3+^-doped fluoride fiber laser with a 3.9-mum emission wavelength. *Appl. Opt.* **36**, 8595–8600 (1997). 18264407 10.1364/ao.36.008595

[14] Zhang, Z. et al. Enhanced 3.9 μm emission from diode pumped Ho^3+^/Eu^3+^ codoped fluoroindate glasses. *Opt. Lett.* **46**, 2031–2034 (2021). 33929411 10.1364/OL.423399

[15] Majewski, M. R. & Jackson, S. D. Numerical design of 4 μm-class dysprosium fluoride fiber lasers. *J. Lightwave Technol.* **39**, 5103–5110 (2021).

[16] Mitschke, F. M. & Mollenauer, L. F. Discovery of the soliton self-frequency shift. *Opt. Lett.* **11**, 659–661 (1986). 19738720 10.1364/ol.11.000659

[17] Gordon, J. P. Theory of the soliton self-frequency shift. *Opt. Lett.* **11**, 662–664 (1986). 19738721 10.1364/ol.11.000662

[18] Lee, J. H. et al. Soliton self-frequency shift: experimental demonstrations and applications. *IEEE J. Sel. Top. Quantum Electron.* **14**, 713–723 (2008). 23055656 10.1109/JSTQE.2008.915526 PMC3465838

[19] Skryabin, D. V. et al. Soliton self-frequency shift cancellation in photonic crystal fibers. *Science* **301**, 1705–1708 (2003). 14500977 10.1126/science.1088516

[20] Koptev, M. Y. et al. Widely tunable mid-infrared fiber laser source based on soliton self-frequency shift in microstructured tellurite fiber. *Opt. Lett.* **40**, 4094–4097 (2015). 26368720 10.1364/OL.40.004094

[21] Li, Z. R. et al. Tunable mid-infrared Raman soliton generation from 1.96 to 2.82 μm in an all-solid fluorotellurite fiber. *AIP Adv.* **8**, 115001 (2018).

[22] Guo, X. H. et al. Dispersive wave generation at 4µm in a dispersion-engineered fluorotellurite fiber pumped by a 1.98µm femtosecond fiber laser. *Optical Mater. Express* **12**, 634–642 (2022).

[23] Duval, S. et al. Watt-level fiber-based femtosecond laser source tunable from 2.8 to 3.6 μm. *Opt. Lett.* **41**, 5294–5297 (2016). 27842116 10.1364/OL.41.005294

[24] Tang, Y. et al. Generation of intense 100 fs solitons tunable from 2 to 4.3 μm in fluoride fiber. *Optica* **3**, 948–951 (2016).

[25] Gauthier, J. C. et al. Femtosecond tunable solitons up to 4.8 µm using soliton self-frequency shift in an InF_3_ fiber. *Sci. Rep.* **12**, 15898 (2022). 36151236 10.1038/s41598-022-19658-8 PMC9508244

[26] Bei, J. F. et al. Experimental study of chemical durability of fluorozirconate and fluoroindate glasses in deionized water. *Optical Mater. Express* **4**, 1213–1226 (2014).

[27] Wu, T. Y. et al. Ultra-efficient, 10-watt-level mid-infrared supercontinuum generation in fluoroindate fiber. *Opt. Lett.* **44**, 2378–2381 (2019). 31042227 10.1364/OL.44.002378

[28] Boyer, L. L. et al. Multiphonon absorption in ionic crystals. *Phys. Rev. B* **11**, 1665–1680 (1975).

[29] Hollenbeck, D. & Cantrell, C. D. Multiple-vibrational-mode model for fiber-optic Raman gain spectrum and response function. *J. Optical Soc. Am. B* **19**, 2886–2892 (2002).

[30] Henderson-Sapir, O., Beniwal, D. & Ottaway, D. Numerical optimization of high power 3.5 μm erbium-doped mid-infrared fiber laser and amplifiers. In *Proceedings of SPIE 11981, Fiber Lasers XIX: Technology and Systems*, 1198116 (SPIE, 2022)

[31] Xiao, X. et al. Theoretical modeling of 4.3 μm mid-infrared lasing in Dy^3+^-doped chalcogenide fiber lasers. *IEEE Photonics J.* **10**, 1–11 (2018).

[32] Chen, H. et al. Experimental and numerical investigation of mid-infrared laser in Pr^3+^-doped chalcogenide fiber. *Chin. Phys. B* **28**, 024209 (2019).

[33] Boutarfaia, A. & Poulain, M. New stable fluoroindate glasses. *Solid State Ion.* **144**, 117–121 (2001).

