# Supplementary material for: Generation of tunable Raman soliton and dispersive wave beyond 4 μm in centimeter-length fluorotellurite fibers
Source: Light Sci Appl. 2025 Sep 24;14:340. doi: 10.1038/s41377-025-02045-z (PMC12457626; doi:10.1038/s41377-025-02045-z)
Supplement: Supplementary file 1 — Supplementary Information for Generation of Tunable Raman Soliton and Dispersive Wave Beyond 4 μm in Centimeter-Length Fluorotellurite Fibers [file 41377_2025_2045_MOESM1_ESM.docx]

Supplementary Information for

**Generation of Tunable Raman Soliton and Dispersive Wave Beyond 4 μm in Centimeter-Length Fluorotellurite Fibers**

Juan Wang^1^, Shunbin Wang^2^, Xiabing Zhou^3^, Mo Liu^1^, Hao Wu^1^, Yu Yin^1^, Zhipeng Qin^3^, Guoqiang Xie^3, *^, Zhenrui Li^1, *^, Pengfei Wang^4,5, *^ and Yichun Liu^4,5^

*Correspondence: Zhenrui Li (lizr20@hrbeu.edu.cn) or Pengfei Wang (pfwang@nenu.edu.cn) or Guoqiang Xie (*[*xiegq@sjtu.edu.cn*](mailto:xiegq@sjtu.edu.cn)*)*

*^1^College of Physics and Optoelectronic Engineering, Harbin Engineering University, Harbin 150001, China*

*^2^Qingdao Innovation and Development Center, Harbin Engineering University, Qingdao 266400, China*

*^3^School of Physics and Astronomy, Key Laboratory for Laser Plasmas (Ministry of Education), Collaborative Innovation Center of IFSA (CICIFSA), Shanghai Jiao Tong University, Shanghai 200240, China*

*^4^School of Physics, State Key Laboratory of Integrated Optoelectronics, Northeast Normal University, Changchun 130022 China*

*^5^School of Physics,* *Key Laboratory of UV-Emitting Materials and Technology, Northeast Normal University, Changchun 130022 China*

*These authors contributed equally: Juan Wang, Shunbin Wang, Xiabing Zhou*

**e-mail：**

Juan Wang: [wjuan96@foxmail.com](mailto:wjuan96@foxmail.com)

Shunbin Wang: [shunbinwang@hrbeu.edu.cn](mailto:shunbinwang@hrbeu.edu.cn)

Xiabing Zhou: [xb_zhou@sjtu.edu.cn](mailto:xb_zhou@sjtu.edu.cn)

Mo Liu: [liumo@hrbeu.edu.cn](mailto:liumo@hrbeu.edu.cn)

Hao Wu: [hwu@hrbeu.edu.cn](mailto:hwu@hrbeu.edu.cn)

Yu Yin: [yy963541@163.com](mailto:yy963541@163.com)

Zhipeng Qin: [lorance1205@sjtu.edu.cn](mailto:lorance1205@sjtu.edu.cn)

Guoqiang Xie: [xiegq@sjtu.edu.cn](mailto:xiegq@sjtu.edu.cn)

Zhenrui Li: [lizr20@hrbeu.edu.cn](mailto:lizr20@hrbeu.edu.cn)

Pengfei Wang: [pfwang@nenu.edu.cn](mailto:pfwang@nenu.edu.cn)

Yichun Liu: [ycliu@nenu.edu.cn](mailto:ycliu@nenu.edu.cn)

**S1. Details for the calculation of Raman response function.**

The calculation of the Raman response function utilizes the intermediate broadening model developed by Hollenbeck and Cantrell^S1^, which represents a significant advancement over simplified single-Lorentzian approximations.

First, we measured the Raman spectrum of the TBAY glass sample by using a HORIBA LabRAM Odyssey spectrometer in the range of 100-1500 cm^-1^. The TBAY glass sample is excited with a 633 nm laser. A Raman spectrum of silica glass is also measured at the same conditions and is used as a standard for correcting errors due to reflectivity and angel of data collection. The Raman gain spectrum of TBAY glass pumped at 633 nm is calculated by a comparison method (with that of silica) and using the following equation^S2^:

$G=\frac{\sigma_{T}\lambda_{s}^{3}}{c^{2}hn^{2}[N(\omega,T)+1]}$ (S1)

where *σ_T_* is the corrected scattering cross section at *T(K)*, *λ_s_* is the Stokes wavelength, *c* is the velocity of light, *h* is the Plank’s constant (6.626×10^-34^ J·s), *n* is the refractive index, *N* is the number density of molecules. The Raman gain coefficient of silica at 633 nm is taken as 1.56×10^−13^ m/W^S2^. The calculated Raman gain spectrum of the TBAY glass is shown in Fig. S1. The intermediate-broadening model is implemented the multi-component analytical model based on convolutions of Lorentzian and Gaussian line shapes. This approach employs seven distinct vibrational modes positioned at 193.6, 349.7, 502.4, 665.5, 701.7, 793.3 and 810.7 cm^-^¹, each characterized by specific amplitudes, Gaussian linewidths, and Lorentzian linewidths.


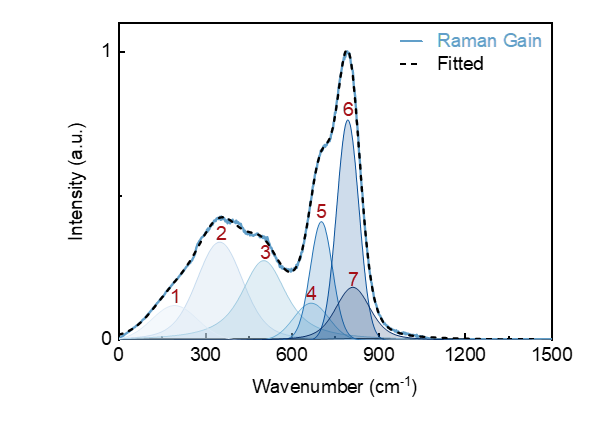


**Fig. S1** Calculated Raman gain spectrum and fitted curve of the TBAY glass with 7 peaks fitted by the intermediate-broadening model

The Raman response function of intermediate-broadening model follows the analytical expression^S1^:

$h_{R}(t)=\sum_{i=1}^{7} \frac{A_{i}^{'}}{\omega_{v,i}}\exp(-\gamma_{i}t)\exp(\frac{-\Gamma_{i}^{2}t^{2}}{4})\sin(\omega_{v,i}t)\theta(t)$ (S2)

where *Aᵢ'* represents modal amplitudes, *ωᵥ,ᵢ* are vibrational frequencies, *γᵢ* and *Γᵢ* denote Lorentzian and Gaussian FWHM respectively, and *θ(t)* is the unit step function. This intermediate-broadening model simultaneously fits both the Raman gain spectrum and response function, providing superior accuracy compared to purely homogeneous or inhomogeneous models.

While originally developed for fused silica, this model has demonstrated excellent transferability to other oxide glass systems including tellurites. Fig. S2 shows the calculated Raman response function.

**Fig. S2** Calculated Raman response function of TBAY fiber

The single-damped-harmonic-oscillator approximation models of the Raman response function is also used to fit the highest Raman gain peak of the TBAY glass. The Raman response function is given as below^S3^:

$h_{R}(t)=\frac{\tau_{1}^{2}+\tau_{2}^{2}}{\tau_{1}\tau_{2}^{2}}\exp(-t/\tau{}_{2})\sin(t/\tau_{1})$ (S3)

𝜏_1_ and 𝜏_2_ were adjustable parameters used to fit the Raman response function and were taken to be 𝜏_1_ = 7.03 fs and 𝜏_2_ = 65.18 fs.

The imaginary and real part of the transfer function $\tilde{h}_{R}(\Delta\omega)$ is:

 (S4)

 (S5)

The calculated imaginary part and the real part of $\tilde{h}_{R}(\Delta\omega)$ is shown in Fig. S3.

**Fig. S3** Calculated imaginary part and real part of $\tilde{h}_{R}(\Delta\omega)$

The Raman gain spectrum is given by^S3^:

$g_{R}(\Delta\omega)=\frac{\omega_{0}}{cn(\omega_{0})}\chi_{\chi\chi\chi\chi}^{(3)}f_{R}\mathrm{Im}[\widetilde{h}_{R}(\Delta\omega)]$ (S6)

where *ω_0_* is the center frequency, 𝛥ω=ω-ω_0_, $Im[\tilde{h}_{R}\left( \Delta\omega\right)]$ is the imaginary part of $\tilde{h}_{R}(\Delta\omega)$. Ignoring the imaginary part of ${\chi_{\chi\chi\chi\chi}}^{(3)}$, ${\chi_{\chi\chi\chi\chi}}^{(3)}$ can be obtained from^S3^:

$n_{2}=\frac{3}{8n}\text{Re}(\chi_{\chi\chi\chi\chi}^{(3)})=3.5\times10^{-19}m^{2}W^{-1}$ (S7)

The fractional contribution of the delayed Raman response *f_R_* can be obtained from Eq. (S6) and (S7). The calculated *f_R_* of the TBAY glass is about 0.32.

**S2. Output repeatability of 4.6 μm Raman soliton.**

To evaluate output repeatability, we conducted systematic reproducibility tests using 3 fiber samples with identical specifications (6.5 μm core diameter, 13 cm length) under consistent coupling and pumping conditions. These 3 samples successfully generated stable 4.6 μm solitons, demonstrating the fundamental reproducibility of our approach at this extended wavelength limit, as illustrated in Fig. S4.

**Fig. S4** Output repeatability of TBAY fibers

**S3.**  **Laser damage characteristics of TBAY fiber and InF_3_ fiber**

The laser damage threshold of TBAY fiber and InF_3_ fiber are investigated. Within the available 3.54 nm pump power range, the TBAY fiber end face is not damaged and the maximum peak power incident on the fiber end face is 1154 kW, corresponding a pulse energy of 260 nJ. Due to the lack of higher-power laser sources operating at 3.54 μm, we employed a 20 W 2 μm continuous wave (CW) laser to evaluate the damage threshold. The evaluation is performed by incrementally increasing the pump power until visible surface damage is observed at the fiber input end. Figure S5(a) shows the experimental setup for high power 2 μm laser delivery. The pump source is a 20 W 2 μm continuous wave (CW) laser. A 10 cm long TBAY or InF_3_ fiber is used for 2 μm laser delivery. The 2 μm is launched into the TBAY or InF_3_ fiber through a couple of aspheric lenses. The output power of the transmitted light is directly measured from the output end of the TBAY or InF_3_ fiber by using a power meter. Figure S5(b) shows the photograph of the InF_3_ fiber before the damage. Figure S5(c) shows the InF_3_ fiber end face after damage by a 2 μm pump laser at 12.3 W. For the TBAY fiber, the measured output power is about 20 W for a launched power of ~17.9 W, no obvious damage is observed on the end surface of the fluorotellurite fiber for more than 1 h.


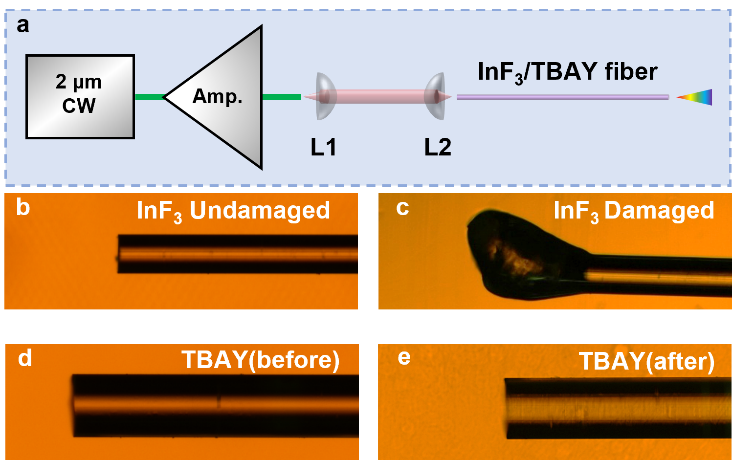


**Fig. S5** **a.** The experimental setup for TBAY fiber and InF3 fiber damage threshold measurement. **b.** The photograph of the InF_3_ fiber before the damage **c.** The photograph of the InF3 fiber after the damage **d.** The photograph of the TBAY fiber before laser delivery **e.** The photograph of the TBAY fiber after laser delivery

**S4.** **Hyperbolic secant function-based optical soliton spectrum fitting**

In the Raman soliton self-frequency shift (SSFS) experiment, hyperbolic secant (sech²) fitting of spectral peaks from temporal soliton pulses revealed high consistency (R^2^> 0.99) across three characteristic peaks (3620 nm, 4025 nm, and 4584 nm), aligning with the fundamental soliton solution of the nonlinear Schrödinger equation. This outcome exhibits fundamental distinctions from supercontinuum generation (SCG) signatures: the measured full-width at half-maximum (FWHM) of soliton peaks remained stably confined to a narrow ~80 nm bandwidth (versus SCG's typical >100 nm broadening), while the central wavelength demonstrated linear redshift with propagation distance—collectively confirming the single-mechanism dominance of SSFS. These results verify successful suppression of SCG-characteristic nonlinear effects (Cross-Phase Modulation, Modulation Instability, Four-Wave Mixing), yielding high spectrally pure controlled Raman solitons, thereby establishing a critical technical pathway for tunable soliton light source design.

**Fig.S6** Hyperbolic secant function-based optical soliton spectral fitting

**References**

[S1] D. Hollenbeck and C. D. Cantrell, Multiple-vibrational-mode model for fiber-optic Raman gain spectrum and response function, J. Opt. Soc. Am. B **19**, 2886-2892 (2002)

[S2] Z. R. Li, Nan Li, C. F. Yao, et, al, Tunable mid-infrared Raman soliton generation from 1.96 to 2.82 μm in an all-solid fluorotellurite fiber. AIP Advances **8** (11): 115001, (2018)

[S3] G. P. Agrawal, Nonlinear Science at the Dawn of the 21st Century. Springer, Berlin, Heidelberg, 195-211. (2000)
